# Stability-Indicating RP-HPLC Method for the Determination of Ambrisentan and Tadalafil in Pharmaceutical Dosage Form

**DOI:** 10.3797/scipharm.1403-22

**Published:** 2014-05-22

**Authors:** Jayvadan K. Patel, Nilam K. Patel

**Affiliations:** ^1^ Nootan Pharmacy College, S. P. Sahkar Vidhyadham, Kamana Crossing, Visnagar 384315, Mehsana, Gujarat, India.; ^2^ Department of Pharmaceutical Sciences, Hemchandracharya North Gujarat University, Patan 384265, Gujarat, India.

**Keywords:** Ambrisentan, Tadalafil, RP-HPLC, Stability-indicating determination, Forced degradation

## Abstract

A simple, rapid, and highly selective RP-HPLC method was developed for the simultaneous determination of Ambrisentan (AMB) and Tadalafil (TADA) drug substances in the fixed dosage strength of 10 mg and 40 mg, respectively. Effective chromatographic separation was achieved using a Hypersil GOLD C18 column (150 mm × 4.6 mm internal diameter, 5 μm particle size) with a mobile phase composed of methanol, water, and acetonitrile in the ratio of 40:40:20 (by volume). The mobile phase was pumped using a gradient HPLC system at a flow rate of 0.5 mL/min, and quantification of the analytes was based on measuring their peak areas at 260 nm. The retention times for Ambrisentan and Tadalafil were about 2.80 and 7.10 min, respectively. The reliability and analytical performance of the proposed HPLC procedure were statistically validated with respect to system suitability, linearity, ranges, precision, accuracy, specificity, robustness, detection, and quantification limits. Calibration curves were linear in the ranges of 1–20 μg/mL for Ambrisentan and 4–80 μg/mL for Tadalafil with correlation coefficients >0.990. The proposed method proved to be selective and stability-indicating by the resolution of the two analytes from the forced degradation (hydrolysis, oxidation, and photolysis) products. The validated HPLC method was successfully applied to the analysis of AMB and TADA in pharmaceutical dosage form.

## Introduction

Ambrisentan (AMB), a non-peptide, is a highly selective endothelin-1 type A receptor antagonist. AMB belongs to the antihypertensive class of drugs and is used in the treatment of pulmonary atrial hypertension in patients with WHO class II or III symptoms. Endothelin is a peptide that constricts blood vessels and elevates blood pressure. AMB blocks the effects of endothelin-1 and thus decreases blood pressure in the lungs. The thickening of blood vessels in the lungs and heart is also inhibited by AMB. AMB is chemically known as (2*S*)-2-[(4,6-dimethylpyrimidin-2-yl)oxy]-3-methoxy-3,3-diphenyl-propanoic acid [1–5].

Tadalafil (TADA), chemically (6*R*,12a*R*)-6-(1,3-benzodioxol-5-yl)-2-methyl-2,3,6,7,12,12a-hexahydropyrazino[1’,2’:1,6]pyrido[3,4-*b*]indole-1,4-dione, is a potent and selective phosphodiesterase-5 (PDE-5) inhibitor, a secondary messenger for the smooth muscle-relaxing effects of nitric oxide, which plays an important role in the vasodilation of erectile tissues [6–8].

To the best of our knowledge, the assay of AMB is not official in pharmacopoeias of India, U.S.P., and BP. The detailed survey of literature revealed that very few methods have been reported for the estimation of AMB alone [9–11]. Tadalafil can be determined in biological samples, dietary supplements, or herbal matrices by liquid chromatography-tandem mass spectrometry with electrospray ionization [12–14], micellar electrokinetic capillary chromatography [15], HPLC [16–23], SIAM HPLC [24, 25], and spectro– photometry [26–28] alone and with other drug combinations.

Ambrisentan and Tadalafil with doses of 10 mg and 40 mg, respectively, were used in combination for pulmonary arterial hypertension under the phase 3 study [29, 30]. No single combination of AMB with any drug is available on the market. No chromatographic method or stability study was published for the combination. Due to the vital significance of AMB and TADA, the development of a sensitive, simple, and fast method for its quantification is of significant need. So, the aim of the present work was to develop a simple, sensitive, accurate, and precise SIAM HPLC in a laboratory-prepared synthetic tablet mixture in the presence of excipients, not a product from the market or a clinical study. The proposed method was validated according to ICH guidelines [31]. The developed method will have significant value for the simultaneous determination of AMB and TADA in their future (or potential) compound formations, and in the body (e.g. blood).

## Result and Discussion

### Method Development and Optimization

The main objective prior to the development of a proper RP-HPLC method was to separate AMB and TADA from the formulation excipients and all degradation products. Moreover, the method should be simple enough for use in a routine quality control laboratory. Various mobile phases have been examined to achieve these specific goals. Both AMB and TADA (10 μg/ml each) spectra have sufficient absorption at 260 nm, which was therefore chosen for the entire study.

**Fig. 1.**
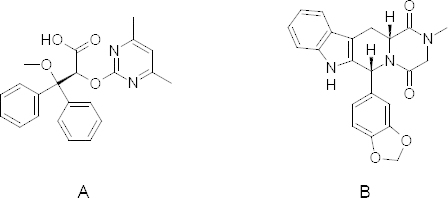
Chemical structure of Ambrisentan (A) and Tadalafil (B)

**Fig. 2.**
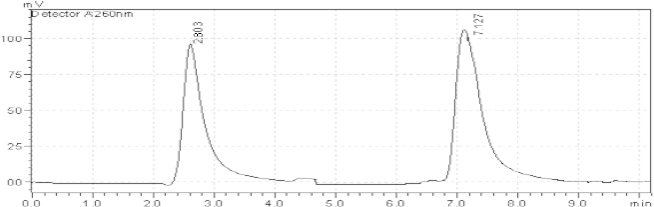
Typical chromatogram of a standard mixture of 10 μg/ml AMB (2.803 minutes), 40 μg/ml TADA (7.127 minutes)

For the study, 10 μg/ml of Ambrisentan and 40 μg/ml of Tadalafil solution were prepared for the entire standard. Different columns like the Hypersil GOLD C18 column (150 mm × 4.6 mm internal diameter, 5 μm particle size), Kromasil 100 C8 column (50 mm × 4.6 mm internal diameter, 5 μm particle size), and ACE 5 C18 column (150 mm × 4.6 mm internal diameter, 5 μm particle size) were tried for method development. The Hypersil GOLD column gave better results compared to the other columns. In almost every system (with changes in the mobile phase) studied, Ambrisentan and Tadalafil showed retention times greater than 2.80 min and 7.10 min, respectively. Other mobile phase combinations resulted in > 2.80 min and 7.10 min, where the separation of AMB and TADA from each other and from the excipients/degradation products was worse than the optimal conditions. The optimized mobile phase consisted of methanol, water, and acetonitrile in the ratio of 40:40:20 (v/v/v). It was not required to change the pH of the mobile phase, as both drugs gave well-resolved peaks in the optimized mobile phase. Figure 1 shows the chemical structures of Ambrisentan and Tadalafil. Figure 2 also shows a typical HPLC chromatogram of the placebo and the freshly prepared mixture of AMB and TADA using the optimized conditions.

### Method Validation

After the successful optimization of the RP-HPLC method, it was validated in accordance to the ICH guidelines. Parameters such as system suitability, specificity (placebo and forced degradation interferences), sensitivity (LOD and LOQ), linearity, range, accuracy (recovery), precision (repeatability and intermediate precision), robustness, and stability-indicating capability were all validated.

### System Suitability

The system suitability was determined by injecting six replicates of the standard solutions and analyzing each active ingredient for its peak area, peak tailing factor, resolution, number of theoretical plates, and capacity factor. The system suitability results for a combined solution of 10 μg/ml AMB and 40 μg/ml TADA revealed a %RSD of less than 1.0% for both peak areas. This method meets the accepted requirements as shown in Table 1.

**Tab. 1.** Summary of the accepted system suitability requirements

Parameter	AMB	TADA	Accepted limit
% RSD	0.40	0.35	≤ 20.0%
Tailing factor (Tf)	0.98	0.96	≤ 2.0
Resolution (Rs)	4.259	–	≥2.0
Number of theoretical plates (N)	4720	3521	≥3000
Capacity factor (k’)	3.63	2.50	≥1.0

### Specificity (Placebo and Forced Degradation Interference)

Generally, the specificity of a method is its suitability for the analysis of a compound in the presence of potential impurities. The placebo, standards, and sample test solutions were all injected at the same wavelength of 260 nm to demonstrate the specificity of the optimized method. A comparison of the retention times of AMB and TADA in sample solutions and in the standard solutions were exactly the same. Figures 1 and 2 showed that there were no interferences at the retention times for AMB and TADA due to the placebo. Therefore, the proposed method is suitable for the quantification of the active ingredients in a laboratory-prepared tablet mixture.

The specificity of the method for AMB and TADA has been assessed by performing forced degradation studies on the active ingredients separately to indicate the initial results, and also on samples of the tablet formulation. The stress conditions studied were sunlight, heat (reflux), acid hydrolysis (1.0 N HCl), base hydrolysis (1.0 N NaOH), and oxidation (30% H2O2). The sample stress solutions were analyzed against the freshly prepared standards and samples. The assay for the stressed standards and sample solutions were calculated as summarized in Table 2. The degradation product is listed in Table 3.

**Fig. 3.**
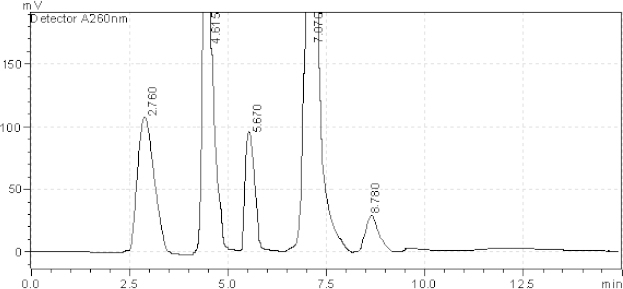
HPLC chromatogram of acidic degradation (1 N HCl) of the tablet solution after reflux for 6 hr, AMB (2.760 minutes), TADA (7.076 minutes). The unknown degraded impurities appeared at 4.615, 5.670, and 8.780 minutes

**Fig. 4.**
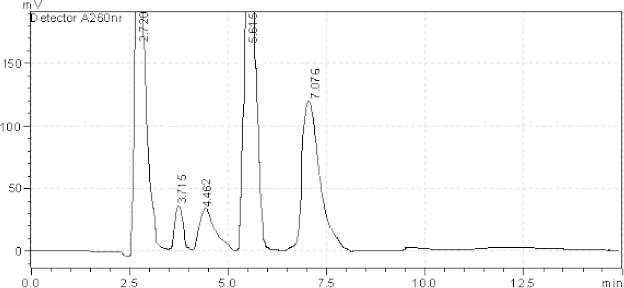
HPLC chromatogram of basic degradation (1 N NaOH) of the tablet solution after RT 24 hr, AMB (2.726 minutes), TADA (7.076 minutes). The unknown degraded impurity appeared at 3.715, 4.462, and 5.615 minutes

**Fig. 5.**
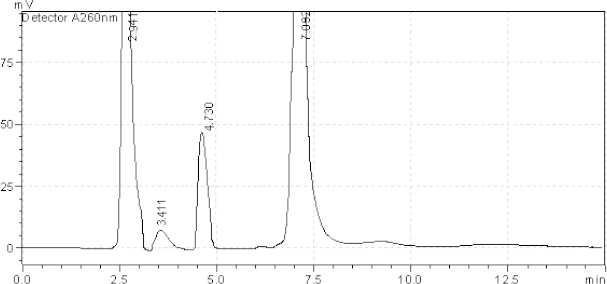
HPLC chromatogram of the tablet solution upon exposure to reflux for 12 hours, AMB (2.941 minutes), TADA (7.092 minutes). The unknown degraded impurity appeared at 3.411 and 4.730 minutes

**Fig. 6.**
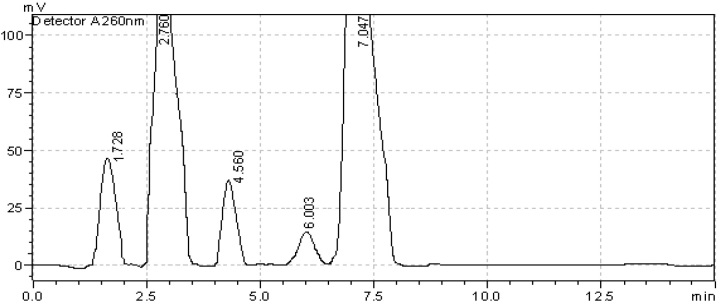
HPLC chromatogram of oxidative degradation of the tablet solution after 3 days, AMB (2.760 minutes), TADA (7.047 minutes). The unknown degraded impurity appeared at 1.728, 2.760, 4.560, and 6.003 minutes

**Fig. 7.**
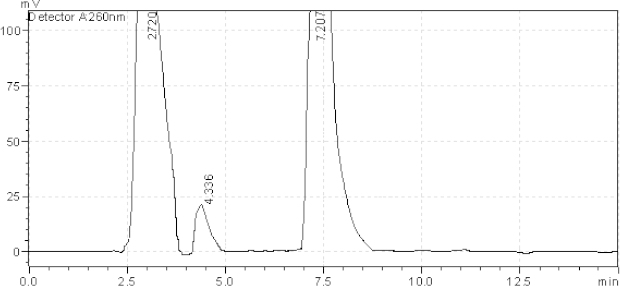
HPLC chromatogram of the tablet solution upon exposure to sunlight for6 hours, AMB (2.720 minutes), TADA (7.207 minutes). The unknown degraded impurity appeared at 4.336 minutes

Table 2 reveals that Ambrisentan showed extensive degradation under acidic and oxidative conditions of degradation. Tadalafil showed extensive degradation under basic conditions of degradation. Resolution for both of the active ingredients was found to be greater than 2.0. Therefore, there was no interference between the main active ingredients and any other stress impurity peaks in the chromatogram. Almost the same pattern of degradation was obtained for both AMB and TADA in their samples. Figures (3–7) show the chromatographic profiles of the active ingredients and the degradation products after exposing the prepared sample solution to different stress conditions as in Table 2.

**Tab. 2.** Summary of the forced degradation of AMB and TADA (API and tablet solution)

Condition of forced degradation	% Degradation of API		% Degradation of formulation	
	AMB	TADA	AMB	TADA
1 N HCI, reflux, 6 hours	54.25	9.30	55	10.26
1 N NaOH, RT, 24 hours	5.30	85	7.85	82.20
Water, 12 hour reflux	16	0	18.20	0
30% w/v H_2_O_2_, 3 days	38.21	16.38	40.29	17.40
Sunlight (6 hr, summer)	5	0	4.92	0

API…active pharmaceutical ingredient.

**Tab. 3.** Summary of the forced degradation product of AMB and TADA standards

Condition of forced degradation	Ambrisentan		Tadalafil	
	RT of Drug	RT of Degradation Products	RT of Drug	RT of Degradation Products
1 N HCl, reflux, 6 hours	2.736	4.74, 5.57	7.08	8.66
1 N NaOH, RT, 24 hours	2.74	3.54, 4.67	7.07	5.67
Water, reflux, 12 hour	2.79	3.43, 4.83	7.07	-
30% w/v H_2_O_2_, 3 days	2.78	4.52, 6.20	7.07	1.74
Sunlight (6 hr, summer)	2.74	4.30	7.07	–

RT…retention time.

### Sensitivity

The sensitivity of the method was explored via measurement of the limit of detection (LOD) and limit of quantitation (LOQ) for AMB and TADA at a signal-to-noise ratio of 3 and 10, respectively. The LOD was found to be 0.22 and 0.98 μg/ml for AMB and TADA, respectively. The LOQ was found to be 0.67 and 2.96 μg/ml for AMB and TADA, respectively, with an RSD of less than 2% (accepted value is less than 10%).

### Linearity

**Fig. 8.**
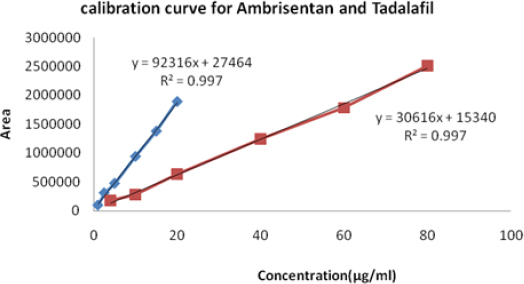
Linearity and range for AMB and TADA

Calibration curves were plotted over the concentration range of 1-20 μg/ml for Ambrisentan and 4-80 μg/ml for Tadalafil. Accurately measured working standard solutions of Ambrisentan (0.01, 2.5, 0.5, 1.0, 1.5, and 2.0 ml) and Tadalafil (0.40, 1.0, 2.0, 4.0, 6.0, and 8.0 ml) were transferred to a series of 10-ml volumetric flasks and the volume in each flask was adjusted to 10 ml with the mobile phase. The resulting solutions were injected into the column and the peak area obtained at retention times 2.80 and 7.10 minutes at a flow rate of 0.5 ml/min were measured at 260 nm for Ambrisentan and Tadalafil, respectively. Calibration curves were constructed by plotting the peak area versus concentration. Each reading was the average of three determinations (Figure 8). Regression analysis data is shown in Table 4.

**Tab. 4.** Regression statistics

Active ingredient	Linearity range (μg/ml)	(R2)	Linearity equation*
AMB	1-20	0.997	Y = 92316X + 27464
TADA	4-80	0.997	Y = 30616X + 15340

* Y is the peak area and X is the concentration.

### Accuracy (Recovery)

Accuracy was determined by the recovery study of known amounts of AMB and TADA standards added to a sample solution. Different concentrations of the two active ingredients were added to the sample and the recovery was measured. The data obtained for the evaluation of linearity were used. The accuracy as reflected from the recovery data and statistical evaluation of the assay for the two active ingredients is listed in Table 5. The average recovery data of AMB and TADA showed results between 99.04% and 100.78%, with the %RSD less than 1.78%, which are within acceptable limits (98.0 to 102.0% recovery and %RSD of no more than 2.0%).

**Tab. 5.** Average recoveries, % RSD values at five concentration levels of spiking AMB and TADA

Drug	Amount taken(μg/ml)	Amount added(μg/ml)	Amount found (μg/ml) ± SD(n=3)	% Recovery ± SD(n=3)
		50%	2.50 ± 0.04	100.20± 1.60
Ambrisentan	5	100%	5.04 ± 0.03	100.78 ± 0.57
		150%	7.50 ± 0.13	99.98 ± 1.78
		50%	9.90 ± 0.04	99.04 ± 0.35
Tadalafil	20	100%	19.97 ± 0.21	99.87 ± 1.60
		150%	29.76 ± 0.29	99.21 ± 0.62

### Precision

Repeatability

The method precision of the instrument was checked by repeatedly injecting (n=6) the standard solution. The assay results and statistical evaluation for the assay of the two active ingredients showed %RSD values of 0.31% and 0.39% for AMB and TADA, respectively, which are within the acceptable limit of 2.0%.

Intermediate Precision (Ruggedness)

Intermediate precision was evaluated in terms of intraday and interday precision by analyzing three different concentrated solutions three times on the same day and on different days over the entire concentration range for both drugs. The assay results and statistical evaluation for the assay of the two active ingredients revealed %RSD values of intraday 0.34–0.71% and 0.29–0.60%, interday 0.51–1.92% and 0.51–1.67%, for AMB and TADA, respectively, which are within the acceptable limit of 2.0%.

Robustness

Pre-determined variations were performed under the experimental conditions of the RP-HPLC method to assess its robustness. The variations imposed on the chromatographic method are summarized in Table 6. The modifications include different mobile phase flow rates of (± 0.1 ml/min) and different column temperatures in the range (± 2°C). Different mobile phase compositions (in the range of ± 1 of the nominal value) and wavelength variations (± 1 nm) were also investigated. The %RSD values showed no significant changes in the final assay results of each of the above two ingredients using variations (Table 6).

**Tab. 6.** Robustness testing of the two active ingredients of AMB and TADA

Parameter	Modification	% Recovery Ambrisentan	± SD (n=6)Tadalafil
Flow rate (0.5 ml/min)	± 0.1	99.49±1.19	100.66±0.92
Mobile phase composition Methanol: acetonitrile water (40:40:20 v/v)	± 1	99.08±1.20	100.55±1.03
Wavelength (260 nm)	± 1	99.26±1.22	100.90±1.06
Injection volume (20 μl)	± 1	99.13±1.07	100.90±1.22
Column temperature (40°C)	± 2	99.81±1.09	101.10±1.12

### Applicability of the Method to the Synthetic Market Product

It is evident from the results obtained that the validated method gave satisfactory results with respect to the analysis of both drugs. The validated method was applied to a laboratory-prepared synthetic tablet mixture as shown in Table 7.

This acceptable value indicated the applicability of the proposed method for the routine quality control of the tablet formulation without interference from the excipients. This was evidenced by the good labeled claim percentages as well as the absence of any peaks in the chromatogram of the placebo.

**Tab. 7.** Results of the synthetic market product

Ambrisentan			Tadalafil		
Label claim (mg)	Amount found (mg) ± SD (n=3)	% Assay ± SD (n=3)	Label claim (mg)	Amount found (mg) ± SD (n=3)	% Assay ± SD (n=3)
5	4.99 ± 0.07	99.89 ± 1.39	20	19.88 ± 0.15	99.42 ± 0.73
5	4.97 ± 0.03	98.25 ± 0.79	20	20.12 ± 0.29	98.52 ± 0.33
5	5.06 ± 0.10	100.28 ± 1.20	20	19.85 ± 0.35	100.52 ± 1.20

## Experimental

### Materials

Reference standards of Ambrisentan and Tadalafil were purchased from Chitichem, Rajkot. HPLC grade methanol, acetonitrile, solvents, water, hydrochloric acid fuming (37%), sodium hydroxide pellets, and hydrogen peroxide (30%), were purchased from Merck (Germany). The synthetic mixture of Ambrisentan and Tadalafil was prepared in the laboratory.

### HPLC System

Gradient high-pressure liquid chromatographies (Shimadzu LC-2010C HT) with the variable wavelength programmable UV/Vis detector, Shimadzu (Kyoto, Japan) model were used for HPLC analysis. The UV-1800 double beam UV-visible spectrophotometer attached with the computer-operated software UV probe 2.0 with a spectral width of 2 nm, wavelength accuracy of 0.5 nm, and pair of 1 cm matched quartz cells was used to measure the absorbance of the resulting solutions. The Sartorius CP224S analytical balance (Gottingen, Germany) and ultrasonic cleaner (Frontline FS 4, Mumbai, India) were used during the study.

### Chromatographic Conditions

The HPLC experimental conditions were optimized on the Hypersil GOLD C18 column (150 mm × 4.6 mm internal diameter, 5 μm particle size) that was purchased from ACE, United Kingdom. The optimum mobile phase was prepared by mixing methanol, water, and acetonitrile in the ratio of 40:40:20 (by volume). The mobile phase was filtered by using a 0.45 μn microporous filter and was degassed by sonication prior to use. A wavelength of 260 nm was chosen since it was found to be the most appropriate for the determination of the two active ingredients because both of the drugs have sufficient absorption at this wavelength. The flow rate used was 0.5 ml/min. The injection volume was 20 μl and the temperature of the column was 40°C. The total run time of the system was about 10 minutes.

### Preparation of the Standard Solution

The standard solution for both drugs was prepared by dissolving 50 mg AMB and TADA reference standards into 20 ml of methanol in two separate 50-ml volumetric flasks. The final volumes were adjusted with methanol to prepare 1000 μg/ml standard stock solutions of both drugs. Using a volumetric pipette, 10 ml of this solution was transferred to a 100-ml volumetric flask and completed to the volume using the mobile phase. The obtained final solution contained 100 μg/ml of AMB and TADA.

### Preparation of the Sample Solution

Twenty tablets of Ambrisentan and Tadalafil were weighed and powdered, separately. An accurately weighed quantity of the powder equivalent to 10 mg of Ambrisentan and 40 mg of Tadalafil was taken into the 100-ml measuring flask and dissolved in 20 ml methanol with sonication. The solution was filtered through a 0.45 μm membrane filter and the residues were washed thoroughly with methanol. The filtrate and washings were combined in a 100-ml volumetric flask and diluted to the mark with methanol to get a final concentration of 100 μg/ml of Ambrisentan and 400 μg/ml of Tadalafil. For the final test solution of Ambrisentan and Tadalafil, 1.0 ml of filtrate of the sample solution was transferred to a 10-ml volumetric flasks and diluted up to the mark with the mobile phase.

### Forced Degradation Study

The ICH prescribed stress conditions such as acidic, basic, oxidative, thermal (solid heat), and photolytic stresses, which were carried out.

### Standard Drug Stock Solutions

Forced degradation studies for both the drugs were carried out under the conditions of hydrolysis, oxidation, and photolysis. An accurately weighed quantity of the powder, 100 mg of Ambrisentan and 400 mg of Tadalafil, was taken into a 50-ml measuring flask and diluted to the mark with methanol to get a final concentration of 1000 μg/ml of Ambrisentan and 4000 μg/ml of Tadalafil. These stock solutions were used for the forced degradation study.

### Acid Hydrolysis

Forced degradation in acidic media was performed by taking 10 ml of the stock solutions of Ambrisentan and Tadalafil, each in separate amber round-bottom flasks. Then 10 ml of 1 N HCl was added and these mixtures were kept at reflux for 6 hours. This solution was neutralized with 1 N NaOH before analysis.

### Base Hydrolysis

Forced degradation in basic media was performed by taking 10 ml of stock solutions of Ambrisentan and Tadalafil, each in separate amber round-bottom flasks. Then 10 ml of 1 N NaOH was added and these mixtures were kept at RT for 24 hours. This solution was neutralized with 1 N HCL before analysis.

### Oxidative Hydrolysis

Degradation with hydrogen peroxide was performed by taking 10 ml of stock solutions of Ambrisentan and Tadalafil, each in two different flasks and adding 10 ml of 30% (w/v) hydrogen peroxide in each of the flasks. These mixtures were kept for up to 3 days in the dark.

### Thermal Degradation

To study neutral degradation, 10 ml of the stock solutions of Ambrisentan and Tadalafil were taken in two different flasks, then 10 ml of HPLC grade water was added in each flask and these mixtures were heated for 12 hr at reflux in the dark.

### Photodegradation

The photostability was studied by exposing the solid state of both drugs to direct sunlight in summer days for 6 hr on a wooden plank.

For HPLC analysis, all the degraded sample solutions were diluted with the mobile phase to obtain a final concentration of 20 μg/ml of Ambrisentan and 80 μg/ml of Tadalafil. Besides, the solutions containing 20 μg/ml of Ambrisentan and 80 μg/ml of Tadalafil were also prepared separately without performing the degradation of both the drugs. Then 20 μl solution of the above solutions were injected into the HPLC system and analyzed under the chromatographic analysis conditions described earlier.

### Forced Degradation Study on the Sample Solution

Twenty tablets of Ambrisentan and Tadalafil were weighed and powdered, separately. An accurately weighed quantity of the powder equivalent to 100 mg of Ambrisentan and 400 mg of Tadalafil was taken into a 100-ml measuring flask and dissolved in methanol with sonication for 20 minutes. The solution was filtered through a 0.45 μm membrane filter and the residues were washed thoroughly with methanol. The filtrate and washings were combined in a 100-ml volumetric flask and diluted to the mark with the mobile phase to get a final concentration of 1000 μg/ml of Ambrisentan and 4000 μg/ml of Tadalafil.

The following conditions for forced degradation were the same as that used for the standard solution. After that, the above solutions were diluted with the mobile phase to get a final concentration of 20 μg/ml of Ambrisentan and 80 μg/ml of Tadalafil. Then 20 μl solution of the above solutions were injected into the HPLC system and analyzed under the chromatographic analysis conditions described earlier.

## Conclusion

The validated HPLC method developed for the quantitative quality control determination of AMB and TADA in combination was evaluated for system suitability, specificity, sensitivity, linearity, range, accuracy (recovery), precision (repeatability and intermediate precision), and robustness. All the validation results were within the allowed specifications of the ICH guidelines. The developed method has proven to be rapid, accurate, and stability-indicating for the simultaneous determination of the combined AMB and TADA in the synthetic tablet mixture in the presence of excipients and the degradation products. There was always a complete separation of both ingredients from their degradation products and from the placebo. As a result, the proposed HPLC method could be adopted for the quantitative quality control and routine analysis of the tablet dosage form or any other formulation.
